# Decision Support Tools in Adult Long-term Care Facilities: Scoping Review

**DOI:** 10.2196/39681

**Published:** 2022-09-06

**Authors:** Linda Lapp, Kieren Egan, Lisa McCann, Moira Mackenzie, Ann Wales, Roma Maguire

**Affiliations:** 1 Department of Computer and Information Sciences University of Strathclyde Glasgow United Kingdom; 2 Digital Health & Care Innovation Centre Glasgow United Kingdom

**Keywords:** decision support, care home, nursing home, digital health

## Abstract

**Background:**

Digital innovations are yet to make real impacts in the care home sector despite the considerable potential of digital health approaches to help with continued staff shortages and to improve quality of care. To understand the current landscape of digital innovation in long-term care facilities such as nursing and care homes, it is important to find out which clinical decision support tools are currently used in long-term care facilities, what their purpose is, how they were developed, and what types of data they use.

**Objective:**

The aim of this review was to analyze studies that evaluated clinical decision support tools in long-term care facilities based on the purpose and intended users of the tools, the evidence base used to develop the tools, how the tools are used and their effectiveness, and the types of data the tools use to contribute to the existing scientific evidence to inform a roadmap for digital innovation, specifically for clinical decision support tools, in long-term care facilities.

**Methods:**

A review of the literature published between January 1, 2010, and July 21, 2021, was conducted, using key search terms in 3 scientific journal databases: PubMed, Cochrane Library, and the British Nursing Index. Only studies evaluating clinical decision support tools in long-term care facilities were included in the review.

**Results:**

In total, 17 papers were included in the final review. The clinical decision support tools described in these papers were evaluated for medication management, pressure ulcer prevention, dementia management, falls prevention, hospitalization, malnutrition prevention, urinary tract infection, and COVID-19 infection. In general, the included studies show that decision support tools can show improvements in delivery of care and in health outcomes.

**Conclusions:**

Although the studies demonstrate the potential of positive impact of clinical decision support tools, there is variability in results, in part because of the diversity of types of decision support tools, users, and contexts as well as limited validation of the tools in use and in part because of the lack of clarity in defining the whole intervention.

## Introduction

### Background

The COVID-19 pandemic has exerted unprecedented pressure on our health and social care infrastructures. It has shown the value of rapid clinical decision-making for improving health and wellness outcomes, particularly for people in vulnerable groups (eg, older adults in care home settings). Despite the considerable potential of digital health approaches to help with continued staff shortages and improve quality of care, digital innovations are yet to make real impacts in the care home sector. There are many barriers to implementation of clinical decision support tools in this sector, including insufficient staff and resources in care facilities, lack of carer time and knowledge [1], and limited use of electronic health records [2].

A number of reviews have been published recently regarding clinical decision support systems in long-term care facilities [2-5]. A scoping review by Abtellatif et al [2] analyzed clinical decision support systems used for pressure ulcer and malnutrition prevention, drug prescription support, and disease management, and they found 10 systems: 3 (30%) used for pressure ulcer and malnutrition prevention, 2 (20%) for medication review, 3 (30%) for daily drug prescription support, and 2 (20%) for disease management (real-time management of heart failure and management of urinary tract infection). Another systematic review by Marasinghe [5] investigated computerized clinical decision support systems used for improving medication safety. Two other systematic reviews investigated clinical decision support systems for pressure ulcer prevention and management [3,4], with the review by Araujo et al [3] assessing the effects on nurses’ clinical decision-making. In addition, they investigated the factors that influence the use and successful implementation of decision support systems in clinical practice. A review by Mäki-Turja-Rostedt et al [4] explored the effectiveness of the interventions.

However, these reviews either focused only on 1 purpose of the decision support tool (eg, medication management or pressure ulcer prevention) or on a certain aspect of the support tool (eg, effectiveness only or implementation only). Hence, there is a need to better understand current evidence for the use of clinical decision support tools in long-term care facilities such as nursing and care homes. More specifically, a deeper understanding of the purpose of such tools, how these tools have been developed, and what types of data these tools use would be considerably advantageous. Therefore, the aim of this review was to analyze studies that evaluated clinical decision support tools in long-term care facilities based on (1) the purpose and intended users of the tools, (2) the evidence base used to develop the tools, (3) how the tools are used and their effectiveness, and (4) the types of data the tools use. It is anticipated that this review will contribute to existing scientific evidence to inform a roadmap for digital innovation, specifically for clinical decision support tools, in long-term care facilities.

In this review, we define *clinical decision support* as described by Greenes [6]: “Clinical decision support tools are aids for making decisions using information and communication technologies that bring relevant knowledge regarding the health and wellbeing of a patient.”

### Objectives

The objective of this review was to seek answers to the following research questions (RQs):

- RQ1: What has been the purpose of clinical decision support tools? Which professionals are the intended users?
- RQ2: What evidence base was used to develop the clinical decision support tools?
- RQ3: How are clinical decision support tools used in adult long-term care facilities? What is the effectiveness of these tools?
- RQ4: What types of data do clinical decision support tools use?

To address the aforementioned questions, we undertook a scoping review [7] by reviewing recent literature (from 2010 onward), using several key search terms across 3 electronic databases.

## Methods

### Data Sources and Search Strategy

We conducted our search using a number of key search terms ((“Decision Support”[tiab]) OR (“Clinical Decision-Making”[Medical Subject Headings term])) AND ((“Care Home”[tiab]) OR (“Nursing Home”[tiab])) AND (2010:2021[pdat]) that were applied across 3 electronic databases: PubMed, Cochrane Library, and the British Nursing Index. Articles were included during the search if they were published between January 1, 2010, and July 31, 2021. In addition, references from included papers were screened for potential additional articles.

### Exclusion and Inclusion Criteria

As decision support tools are an emerging area of published literature, we developed inclusion criteria (Table 1) across the parameters of setting, study design, type of decision support, user, and comparator. In terms of study design, only studies that tested or evaluated the decision support tool, as opposed to only developing the tools, were included in the review. In addition, we only included studies in English.

**Figure 1:**
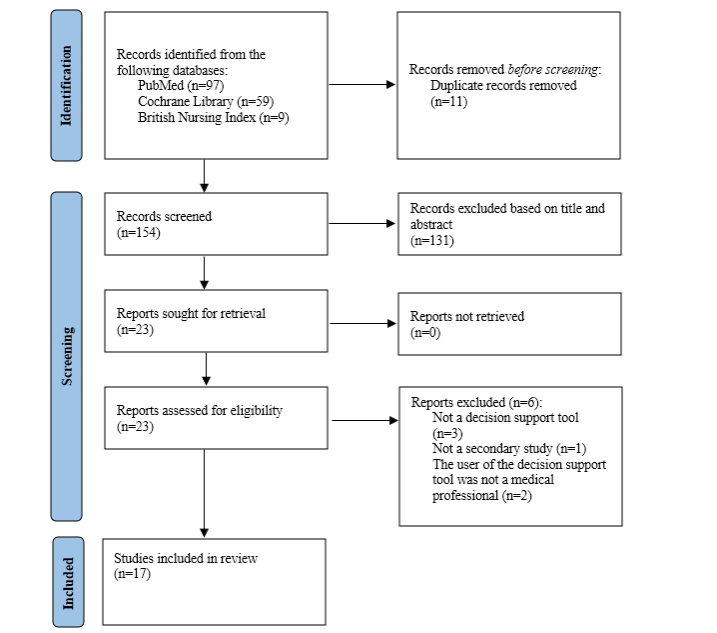
Flowchart of inclusion and exclusion of reviewed papers.

The exact process we used to exclude studies is further explained in the Results section and shown in the PRISMA (Preferred Reporting Items for Systematic Reviews and Meta-Analyses) diagram (Figure 1).

**Table 1:** Inclusion and exclusion criteria for papers based on setting, study design, and type of decision support.

Variable	Inclusion criteria	Exclusion criteria
Setting	Adult long-term care facilities (eg, adult care homes and adult nursing homes)	Nonadult long-term care facilities (eg, nonadult care homes, hospitals, and short-term care facilities)
Study design	The study is about testing of the decision support tool (eg, feasibility study, evaluation study, randomized controlled trial, or implementation study)	The study is developing a decision support tool without testing the tool (ie, primary study)
Type of decision support	The decision support tool is for patients’ health conditions (both mental and physical health)	The decision support tool is for management purposes (staff planning, bed planning, etc)
User	Health or social care professional	Patient or family member
Study language	English	Other than English

### Screening and Description of Included Studies

We screened all articles identified from the search in terms of title and abstract for potential eligibility. Where identified publications potentially met the inclusion criteria, a full text of the article was obtained for further examination. Data were collated and coded using NVivo 12 (QSR International) and Mendeley reference management software (Mendeley Ltd). Microsoft Excel was used to manage extracted data. A description of the process flow and decisions made was collated through the use of a PRISMA flow diagram [8]. Details extracted from the included publications included the following:

1. Publication details: first author of the study, year of publication, country in which the study was conducted, and scale of intervention
2. Decision support tool details: first author and year of developer publication, condition or purpose for which the tool was developed and tested, intended users of the tool, format of the tool (how it operates; eg, real-time, retrospective, or triaging system), whether it is reported to be linked to the electronic health records, whether it is reported to use a validated clinical decision support tool, and what evidence base was reported to be used to develop the tool
3. Study details: type of study (feasibility study, evaluation study, randomized controlled trial, implementation study, reflective or opinion piece, or case study), study setting, study population, study outcome, and whether there was a significant impact on the outcome from using the tool

These details of the studies have been presented in the data extraction table in Multimedia Appendix 1.

## Results

### Overview

In total, 165 papers were identified for potential inclusion; as shown in Figure 1, the search produced 97 (58.8%) papers from PubMed, 59 (35.8%) from Cochrane Library, and 9 (5.4%) from the British Nursing Index. Of these 165 papers, 11 (6.7%) were duplicates and were removed. Of the remaining 154 papers, based on title and abstract screening, 23 (14.9%) were retrieved for full-text review. Of these 23 papers, 6 (26%) were excluded for the following reasons: not studying a decision support tool (n=3, 50%), not being a study evaluating the tool (n=1, 17%), and the user of the tool not being a health or social care professional but a nursing home resident or a family member (n=2, 33%). Thus, of the 165 papers initially identified for potential inclusion, 17 (10.3%) were included in the final review [9-25].

### Setting and Study Population

All research was undertaken in high-income–country settings, including the United States (5/17, 29%), the United Kingdom (3/17, 18%), Canada (3/17, 18%), Sweden (2/17, 12%), Belgium (1/17, 6%), France (1/17, 6%), Norway (1/17, 6%), and the Netherlands (1/17, 6%).

The study population varied greatly from study to study. In studies involving care or nursing homes residents, the largest study population was 6161 residents [21], and the smallest study population was 52 residents in the study by Walker et al [24]. In studies involving health care professionals, the largest number of participants was 27 staff members in the study by Coulongeat et al [10], and the smallest number of participants was 14 registered nurses in the study by Johansson-Pajala et al [16].

### RQ1: What Has Been the Purpose of Clinical Decision Support Tools? Which Professionals Are the Intended Users?

There were 8 different conditions or purposes supported through the use of clinical decision support tools. They included medication management (5/17, 29%) [12,13,15-17], pressure ulcer prevention (4/17, 24%) [9,11,14,21], dementia management (3/17, 18%) [18-20], falls prevention (3/17, 18%) [21,23,24], hospitalization (2/17, 12%) [21,22], malnutrition prevention (1/17, 6%) [14], urinary tract infection (1/17, 6%) [25], and COVID-19 infection (1/17, 6%) [10].

In total, 65% (11/17) of the studies defined the professional user group using the decision support tool, whereas 35% (6/17) did not indicate who the intended users of the tools were [11,13,17,21,24,25]. Where stated (5/11, 45%), the most commonly specified professionals were either *care home staff* or *nursing home staff* [10,14,18,22,23]. Of these 5 studies, 4 (80%) specified that the clinical decision support tool that was evaluated was used by *nurses*; 50% (2/4) of these studies provided further detail on the types of nursing staff, including *registered nurses*, *special needs educator*, and *nurse aides* [14] or *directors, physicians, and nurses* [10]. Of the 11 studies that defined the professional user group using the decision support tool, 1 (9%) specified that the users of the decision support tool were *pharmacists* [12], and 1 (9%) stated that the tool was used by *health professionals* [16].

### RQ2: What Evidence Base Was Used to Develop the Clinical Decision Support Tools?

Overall, 88% (15/17) of the studies provided further information on how the support tools were developed. Among these 15 studies, 9 (60%) stated that the tools were developed using clinical guidelines [13,15,17,20,25-29], 11 (73%) stated that the decision support tools were developed through users’ opinions [13,17,20,27-33] (eg, through the Delphi method [9,28] or other ways of stakeholder involvement [17,20,27,29,33]), 2 (13%) stated that the support tools were developed using systematic reviews of scientific evidence [34,35], and 2 (13%) used data analysis to understand the factors associated with the investigated outcome [30,32].

Of the 17 included studies, 11 (65%) evaluated the effectiveness of the decision support interventions, but no clear consensus could be arrived at on the association between the effectiveness and involvement of stakeholders or use of clinical guidelines in their development and effectiveness. Of these 11 studies, 2 (18%) evaluating tools that involved stakeholders in their development achieved significantly positive results [9,21], whereas 4 (36%) achieved mixed results [16,17,23,24], and 3 (27%) obtained nonsignificant results if the tools involved stakeholders in the development [11,20,22]. When it came to using clinical guidelines for developing decision support tools, none of the 5 studies concerned reported significant results: 4 (80%) demonstrated mixed results [16,17,23,25], and 1 (20%) obtained nonsignificant results [20].

### RQ3: How Are Clinical Decision Support Tools Used in Adult Long-term Care Facilities? What Is the Effectiveness of These Tools?

#### Medication Management

Of the 17 included studies, 5 (29%) focused on medication management (12,13,15-17).

De Wit et al [12] evaluated a clinical decision support system that was designed for medication management. The system operates by extracting medication data of residents and 2 weeks’ worth of historical laboratory data from electronic health records for all residents. Since 2008, a total of 39 clinical rules have been created in the system. If a laboratory value is deemed to be abnormal, in combination with the appropriate drug, the system generates an alert. The system then helps with dosage adjustments in accordance with various conditions, such as decreased renal function or electrolyte dysfunction [12].

The study showed that only 3 clinical rules had an efficiency of >10% *(phenytoin with hypoalbuminemia*, *bisphosphonates dosage regime*, and *ceftazidime with decreased renal function*). Most of the clinical rules demonstrated efficiencies of <10%, and the efficiency of 2 rules was 0% (*oral oncolytics and stop dates* and *methotrexate dosage regime*). The efficiency was calculated by dividing the number of actions for both new alerts and repeat alerts by the total number of new and repeat alerts [12]. As this was a retrospective analysis of a database, there was no control group. This means that it is difficult to evaluate whether the system had a significant effect on improved medication management.

Dorfman et al [13] investigated the potential benefits of a clinical decision support system identifying drug-gene interactions in nursing home residents who were being treated with multiple medications. They tested the system on 987 residents at 4 nursing homes. The pharmacogenetic (PGx) system uses residents’ medication data and electronic health record information, together with genetic information stored in the electronic health records, to produce information regarding drug-drug interactions and other potentially dangerous drug therapy problems. On the basis of the information in the health record systems and algorithms built into the PGx system, the PGx system offers guidance to nurses and pharmacists. The study concluded that the intervention has the potential to be useful for nurses when obtaining a profile of patients’ medication regarding drug-drug interactions, therapeutic duplications, and warnings for unsuitable drugs [13]. However, this is a qualitative study; therefore, no statistical significance was explored.

Johansson et al [15] evaluated the LIFe-reader, which is a PDA with a mobile medical decision support system, that was developed for safer medication management in nursing homes. The tool is used to scan the European Article Number codes on drug packages, through which the LIFe-reader generates alerts for inappropriate drugs and drug combinations: drug-drug interactions, therapeutic duplications, and warnings for drugs unsuitable for older adults. In addition to the aforementioned features, the tool includes Microsoft Word, Microsoft Excel, email, calendar, calculator, and phone. Through interviewing 22 registered nurses at various care homes, the evaluation study found that the scanning function was easy and time saving, and the LIFe-reader was useful and user friendly. However, the users requested more content and functions on the device [15].

Johansson-Pajala et al [16] studied the use of a web-based computerized decision support system that was designed for drug prescribing and medication reviews. The system is linked to electronic medical records and evaluates the quality of drug treatments based on national indicators and potential adverse drug reactions based on the residents’ symptoms. The system produces warnings and explanations about inappropriate drugs, drug-drug interactions, drug use in decreased renal function, and possible adverse drug reactions. The evaluated system includes 2 widely used criteria: screening tool of older people’s prescriptions, screening tool to alert to right treatment, and Beers criteria.

Kane-Gill et al [17] evaluated a clinical decision support system called TheraDoc, a clinical surveillance system containing predeveloped alerts and customizable alerts to detect potentially inappropriate prescribing, which is integrated into electronic health records. More information can be found on the TheraDoc website [36]. Alerts were created for high-risk medications, laboratory monitoring alerts, and antibiotic-stewardship–related alerts, all developed with the purpose of preventing adverse drug events. The alerts are delivered in real time [17].

The tool is reported to have been developed using medical guidelines and users’ opinions; however, the study does not state exactly who the intended users of the system are. The evaluation was undertaken at 4 nursing homes, with 2127 nursing home residents as participants [17].

Of the aforementioned 5 studies focusing on medication management, only 2 (40%) were comparative studies. The study by Johansson-Pajala et al [16] showed the intervention to report significantly more adverse drug reactions and more drug-drug interactions than registered nurses. There was no significant difference between reports of inappropriate drugs and drug duplications when comparing the intervention with the actions of registered nurses. The study also investigated the nurses’ views on drug management; however, the results were nonconclusive. More specifically, the registered nurses did not find that the decision support system significantly affected their drug management methods; however, many saw potential benefits of using the system [16].

The study by Kane-Gill et al [17] showed that the intervention group had significantly lower incidence of alert-specific adverse drug events than usual care. There was no statistically significant difference between the groups for all-cause hospitalizations and 30-day readmissions [17].

#### Pressure Ulcer Management and Nutrition

Overall, 24% (4/17) of the papers focused on pressure ulcer management and nutrition [9,11,14,21].

Beeckman at al [9] evaluated an electronic clinical decision support system called PrevPlan that generates a resident-tailored protocol for pressure ulcer prevention. After data entry to the system regarding the availability of preventive materials and residents’ characteristics (manual entry), the protocol included recommendations regarding skin observation, the use of support surfaces, repositioning, and heel elevation. The evaluation, which involved 464 nursing home residents and 118 health care professionals, reported that the participants had more positive than negative attitudes regarding the decision support tool, with the difference being statistically significant. It was also found that nurses with specific training regarding pressure ulcer management and higher education levels had more positive attitudes than nurses who were not experts in pressure ulcer management or were in the early years of studying to become nurses. The study found that the experimental group had significantly lower pressure ulcer incidence than the control group [9]. Further information regarding the support system can be found on the PrevPlan website [37]; however, the website was last updated in 2011 and is not available in English.

Fossum et al [14] investigated the Risk Assessment Pressure Sore (RAPS) scale for pressure ulcer risk screening and the Mini Nutritional Assessment (MNA) tool for screening nutritional status.

Both Olsho et al [21] and Davidson et al [11] evaluated *On-Time Pressure Ulcer Prevention*, which uses risk reports embedded in electronic health records to identify recent changes in risks for developing pressure ulcers. Although both studies evaluated the same decision support system, it can be assumed that these studies are not related. The system gathers information on residents’ nutritional status, incontinence issues, and recent pressure ulcer history. The documentation is then used to produce 4 weekly core reports, identifying residents at high risk for pressure ulcer formation, enabling monitoring of weekly changes in risk. On-Time relies on staff communication across disciplines and documentation by certified nursing assistants. A certified nursing assistant is a person who helps patients with activities of daily living and other health care needs under the direct supervision of a registered nurse. A *change team* incorporates the reports from On-Time into clinical workflow and identifies which changes in care are required to manage the risk of developing pressure ulcers [11,21].

Olsho et al [21] carried out the evaluation, which used interrupted time-series design, at 25 nursing homes with 6161 nursing home residents as participants. The study found that the intervention components reduced pressure ulcer incidence individually and in combination [21].

Davidson et al [11] evaluated the system at 47 nursing homes; however, the study did not specify the number of participants. For their evaluation method, Davidson et al [11] used difference-in-differences design and investigated the scalability of the intervention. In spite of the large number of nursing homes involved in the study, the authors did not find whether the tool played a significant role in improving pressure ulcer prevention [11].

Of the 4 studies that investigated clinical decision support systems that aimed to help prevent pressure ulcer formation, 2 (50%) found that the incidence of pressure ulcers significantly decreased in the intervention group [9,21]; however, the remaining 2 (50%) studies found no statistically significant differences in terms of pressure ulcer incidence [11,14]. Only the study by Fossum et al [14] used a validated tool (RAPS) for pressure ulcer management. In their study, Fossum et al [14] did not find significant differences in nutritional status of residents between the intervention and control groups when using the MNA.

#### Dementia Management

In total, 18% (3/17) of the studies focused on evaluating decision support tools developed for dementia management. Keenan et al [18] and Moniz-Cook et al [20] evaluated 2 different decision support systems that aimed to help care home staff support residents with commonly occurring challenging behaviors. The system included assessment tools that collected relevant information regarding the residents and then applied logic-based algorithms that generated biopsychosocial action plans that the staff could implement [18,20].

The study by Keenan et al [18] was a qualitative study looking at the contextual and organizational mechanisms of, as well as barriers and facilitators for, the intervention. Four mechanisms of implementation of the intervention were identified: (1) access to, and use of, care homes; (2) resources in terms of IT for e-learning activity; (3) demonstrating capacity to apply action care planning in care practice; and (4) receptivity of care home staff to e-learning and the individually tailored action care planning that followed [18].

The study by Moniz-Cook et al [20] was a cluster randomized trial undertaken at 63 care homes. In total, 658 nursing home residents and 436 care home staff members took part in the study. It was found that there was no statistically significant difference in the number of incidents of challenging behavior between the intervention and control groups. The intervention did not significantly affect the experience of staff members with regard to the prescription of psychotropic medication. The quality of life of residents was not measured because of the large amount of missing data [20].

Kovach et al [19] evaluated a decision support tool called the Serial Trial Intervention, which aimed to help with assessment and treatment of pain and other physical problems of residents with advanced dementia who are unable to report symptoms clearly or consistently. The tool is a 9-step assessment and treatment process, previously evaluated as a 5-step tool. If an assessment is negative, or if interventions fail to decrease symptoms, the nurse moves to the next step. The study, undertaken at 12 nursing homes with 125 nursing home residents, compared the effectiveness of the 2 versions of the protocol. It found that the residents being treated using the 9-step intervention received more assessment-driven treatment and evaluation-driven follow-up. It was also found that these residents had less static and dismissive care than those treated using the 5-step intervention [19].

#### Falls Prevention

Of the 17 included studies, 2 (12%) focused on evaluations of interventions that aimed to support the management or prevention of falls. Tzeng et al [23] carried out a quality improvement project to evaluate the impact of the Fall Tailoring Interventions for Patient Safety program on preventing falls and fall-related injuries among residents. This program was developed to help staff modify falls prevention interventions based on daily assessments, and it can be used as a personalized falls prevention plan displayed on screens placed at residents’ bedsides. The program was developed to help nursing staff identify evidence-based interventions for each area of risk. In the paper, Tzeng et al [23] report that after implementing the intervention the reduction in the average monthly fall rate was clinically significant: the average monthly fall rate reduced from 10.07 falls to 7.95 falls. However, no statistical significance was reported [23].

Walker et al [24] evaluated the Guide to Action Care Home falls prevention program, which consists of posters and paper-based decision support tools in the form of a checklist that helps to identify risk factors associated with falls and suggests actions to reverse or modify these falls risk factors. Walker et al [24] found that the fall rates were lower, and there were nearly twice as many general practitioner visits at control homes than at intervention homes over 6 months of follow-up. That being said, no statistical significance was reported because of the small number of falls [24].

#### Other Purposes: Hospitalization, Urinary Tract Infection, and COVID-19 Infection

Pasay et al [25] evaluated a decision support tool based on the principles of building a culture of safe, effective, and sustainable antimicrobial use for urinary tract infection. The intervention consisted of 4 parts: education of physicians, nursing staff, families, and caregivers; posters with myths and facts regarding the diagnosis and treatment of urinary tract infection; a pamphlet for family and caregivers; and a clinical tool to help with behavioral changes in residents (drugs, eyes and ears, low oxygen states, infection, retention of urine or stool, ictal, underhydration or undernutrition, metabolic, and subdural [DELIRIUMS] tool). The evaluated decision support tool is a checklist that guides staff to identify urinary tract infections based on clinical symptoms, to collect a urine culture only when indicated, and to review antimicrobial therapy if prescribed. The checklist also acted as an interprofessional communication tool [25].

Pasay et al [25] found that there was a statistically significant reduction in urine testing in the intervention group compared with the control group. There was also a statistically significant reduction in the rates of antimicrobial prescribing in the intervention group compared with the control group. There were no differences in admissions to acute care or the emergency department between the 2 groups [25].

Tena-Nelson et al [22] evaluated a program called Interventions to Reduce Acute Care Transfers New York, which consisted of six parts: (1) the Situation, Background, Assessment, Recommendation (SBAR) tool, which was designed to help with communication among medical professionals; (2) the Early Warning Tool (Stop and Watch), which was designed to help in recognizing significant change in residents early; (3) a hospital transfer review tool to guide retrospective review of hospitalizations; (4) a standardized patient transfer form and a checklist; (5) care paths to guide treatment options for common conditions; and (6) advance care planning tools to guide decision-making and communication about end-of-life care.

SBAR is a structured communication format that enables health information to be transferred between individuals and institutions. It aims to convey critical information understandably, clearly, and succinctly [38].

Stop and Watch is a tool to help spot the signs warning that a person’s condition is deteriorating. The poster helps staff to recognize signs and take steps to reduce a person’s risk of morbidity, further disability, organ failure, and mortality [39].

According to the study by Tena-Nelson et al [22], there were no statistically significant changes in hospitalization rates between before and after the intervention. No statistically significant factors were found to be associated with the changes in hospitalization rate. The authors stipulated that the program’s effectiveness could be improved by including participant recommendations on planning, staff and stakeholder engagement, implementation, training, and sustainability [22].

Coulongeat et al [10] investigated a local support platform that aimed to help nursing homes manage their cases of COVID-19 infection. Although the other decision support tools included in the review consisted mostly of 2 actors (a human and a computer [or paper in some cases]), the COVID-19–infection management tool used multiple human actors as the decision support. The reason for this might be that because COVID-19, at the time of tool development, was a very new disease, there was very little evidence available to support the development of a computer program that could help manage the condition. The decision support aspect consisted of a multidisciplinary team, a specialist phone hotline, and mobile geriatric medicine teams, all reachable through information and communication technologies. The intervention helped to satisfactorily address some issues that were revealed by the COVID-19 pandemic. These issues were as follows: limiting the feeling of isolation, getting the health professionals’ questions answered, providing solutions to individual problems, and reassurance of the nursing home staff regarding the optimal treatments for residents. The intervention was less effective in improving the quality of life for residents or staff at nursing homes with a COVID-19 cluster [10]; however, why this was the case is not explained in the paper.

### RQ4: What Types of Data Do Clinical Decision Support Tools Use?

#### Integration With Electronic Health Records

Of the 17 included studies, only 4 (24%) described decision support tools that were integrated with an electronic health record, all of which were developed for medication management [12,13,16,17]. However, it is not known what these electronic health records consist of, whether these are stand-alone systems for the care facilities, or whether these records are linked with general practices. On the basis of the information presented, it is known that in 12% (2/17) of the included papers, the decision support tools were not linked to any electronic health records because they were paper-based posters [23,24]. For the remaining 65% (11/17) of the studies, it is unknown whether the decision support tools were stand-alone systems or linked to electronic health records.

#### Data Used by Clinical Decision Support Tools

Overall, based on their original studies, it was unclear what kind of information the decision support tools required. In total, 29% (5/17) of the studies were clear about the data being collected to aid decision-making. Of these 5 studies, 2 (40%) described decision support tools used for dementia management (Serial Trial Intervention [19] and DemCare [20]), 3 (60%) described decision support tools used for pressure ulcer prevention and management (On-Time [11,21] and RAPS [14]) as well as malnutrition prevention (MNA [14]), and 1 (20%) described a decision support tool used for urinary tract infection prevention and management (multimodal antimicrobial stewardship intervention [25]).

## Discussion

### Overview

In this review we set out to collate current knowledge within the academic literature focused on decision support tools in long-term care settings. Decision support tools are an emerging area of research and practice spanning a range of different conditions, health, and social care professions. However, many studies to date have focused on small-scale, localized efforts; only 17 studies conducted in only 8 high-income–country settings were found to have evaluated decision support tools developed for use in long-term care facilities. Furthermore, although a small number (3/17, 18%) of the identified studies present favorable outcomes, this was not universally true, and there is often a reliance on early evidence such as short-term evaluation studies and analysis of qualitative data. Thus, although this area of research holds significant potential, our findings suggest that review of the published literature is timely to inform future innovation.

We are now moving to a data-driven health and social care model; therefore, the concept of siloed data needs to be a thing of the past, and available data must be used for the benefit of residents of nursing and care homes and to provide added value.

### Principal Findings

In terms of setting and study sample, the majority (13/17, 76%) of the included studies were local, with only 24% (4/17) being carried out on a national scale. That being said, the majority (15/17, 88%) of the studies were multicenter studies, with only 12% (2/17) [23,40] being single-center studies. In terms of the clinical populations who were using the decision support systems at adult long-term care facilities, the most common were staff members, nurses, pharmacists, and health professionals in general. However, the studies in general did not explicitly specify who the intended users were. In addition, 35% (6/17) of the studies did not indicate who the intended users of the tools were.

It should be noted that it is uncommon for long-term care facilities, such as care homes and nursing homes, for example, in the United Kingdom, to have a physician or pharmacist present. Instead, care facilities have partnerships with local general practitioner practices. Hence, it is unlikely that staff within care or nursing homes will be making decisions about medications [41].

Although there is potential for clinical decision support tools to streamline care services by making them more efficient, the systems have been developed to address issues that often fall under the clinical responsibility of nursing staff; therefore, as expected, most users fall within this profession. However, lack of detail regarding users does limit insights into the implementation of the system and therefore may be deemed to inhibit the transferability and scaling up of these systems to other sites and domains [42].

Considering the average age of long-term care residents, the conditions of focus for the clinical decision support tools are not surprising. There were 8 different conditions or purposes supported using clinical decision support tools: medication management, pressure ulcer prevention, dementia management, falls prevention, hospitalization, malnutrition prevention, urinary tract infection, and COVID-19 infection. It is noteworthy that all tools seem to focus on domains of physical health; none focused on mental health, despite it being widely recognized that rates of anxiety and depression are high in this population [43].

Looking at studies demonstrating evidence of whether there was a significant improvement through using the clinical decision support tools, 71% (12/17) of the studies carried out comparative analyses. Of these 12 studies, only 3 (25%) reported clear significance in the results, showing that the evaluated decision support tools made a difference in either preventing negative outcomes or improving care in general [9,19,21].

Of these 3 studies, 2 (67%) evaluated clinical decision support tools developed for pressure ulcer management [9,21]. Olsho et al [21] and Davidson et al [11] evaluated the same tool for pressure ulcer management; however, Davidson et al [11] reported no statistically significant change in the incidence of pressure ulcers when the intervention was used. According to a systematic review by Mäki-Turja-Rostedt et al [4], there are many ways to prevent pressure ulcer formation in residents of long-term care facilities; however, there is a lack of systematic evidence of the most effective way to do this. In their systematic review, Araujo et al [3] agree and add that clinical effects, such as outcomes in the incidence and prevalence of pressure ulcers, remain limited, and most investigated studies found clinically but nonstatistically significant results in decreasing pressure ulcer incidence. The results from this review are in concordance with the comments made in these systematic reviews [3,4].

Of the 17 studies, 5 (29%) found mixed results, meaning that the intervention improved some outcomes but reported no statistically significant difference in other outcomes [16,17,23-25]. However, it is important to note that 40% (2/5) of these studies mentioned “clinical significance” but did not define how this clinical significance was measured and did not report on statistical significance [23,24]. In total, 24% (4/17) of the studies included in the review showed no statistically significant difference when using the intervention [11,14,20,22]. This lack of definitive evidence underpinning digital health solutions is widely recognized; often, tools are implemented within large organizations with very little evidence underpinning them [2,5]. This lack of evidence underscores the need for robust evaluation of solutions used to identify benefits of, and value of investment in, decision support tools [44].

It is important to note that, as explained previously and shown in the data extraction table (Multimedia Appendix 1), there is a noticeable degree of heterogeneity among the decision support tools described in the included studies in terms of their purposes and intended users. However, even among decision support tools with similar purposes, the study designs and measured outcomes in the included evaluation studies varied substantially. Hence, these studies should be compared with one another with extreme caution. Because of this variability, further studies, using standardized methods to evaluate the decision support tools included in this review, are needed.

### Evidence Base of the Development of Clinical Decision Support Tools

An important question that this review aimed to answer concerns the evidence base underpinning the development of decision support tools. Our findings demonstrate that there is a lack of coherent information about what evidence base was used to develop the clinical decision support tools. Most tools were developed based on current guidelines and stakeholders’ opinions. Some studies used systematic reviews of scientific evidence or data analysis to understand the factors associated with the outcome of the tool. In 12% (2/17) of the studies, there is no information regarding the evidence base of the development of the tools. The lack of transparency in underpinning evidence of development of the tools can affect users’ trust in the tools, ultimately affecting their wider uptake [42].

### Adoption and Implementation of Clinical Decision Support Tools

For successful implementation of clinical decision support tools, educational training and culture change are required to sustain their clinical use. Clinical decision support tools, especially those helping to manage medications, need to be regularly updated with regard to changing guidelines and newly available drugs. Ease of updating is an important factor for being considered a successful decision support tool [2,5,38].

A key consideration in terms of the mechanisms and actions of the clinical decision support tools is the data that they use. Overall, in the included studies, all tools, apart from the paper-based decision support tools (n=2), are computer programs. However, most (11/17, 65%) of the studies were not explicit about whether the computer programs were linked to electronic health records, and if they were linked (4/17, 24%), then what kind of electronic health records they were linked to.

Of the 17 included studies, only 3 (18%) described decision support tools that used validated clinical tools. These validated clinical tools are RAPS and MNA used in the evaluation study by Fossum et al [14]; screening tool of older people’s prescriptions, screening tool to alert to right treatment, and Beers criteria used in the evaluation study by Johansson-Pajala et al [16]; and SBAR and Stop and Watch used in the evaluation study by Tena-Nelson et al [22]. When considering the studies (4/17, 24%) that evaluated clinical decision support tools helping to prevent pressure ulcer formation, the study by Fossum et al [14] was the only one to use validated clinical tools.

Considering how long electronic health records have existed and how long researchers have been working on predictive modeling in health, it is surprising that more widely validated decision support tools were not included in the evaluation studies.

It can be assumed that the evaluated tools were developed keeping in mind that electronic health records are not widely used in long-term care facilities such as care and nursing homes. Most of the tools presented in this review, except for medication management tools, do not necessarily require data that are normally stored in electronic health records (eg, temporary symptoms such as meal intake and urinary frequency). Hence, these tools require manual data input from staff, which requires developing suitable infrastructure for tool use (eg, the availability of computers or tablets) at the facilities and training of, and time from, staff. It has been found that these can affect methodological challenges when validating clinical tools, such as gaining acceptance from stakeholders who have limited time in addition to having to deal with work pressure, such as registered nurses, care home managers, and general practitioners [45].

If long-term care facilities would adopt electronic health records specific to these facilities, the burden of having to manually input data for each different decision support tool would be removed, and the tools can be integrated into the practice automatically. This has been successfully implemented in various hospital settings. The systematic review by Varghese et al [46] provides examples of such systems successfully implemented in hospitals.

It is important to understand, however, that hospitals are data-rich environments with often automated data collection (eg, intensive care units), and the purpose of the constant data collection is due to having to always monitor patients. In addition, it is worth noting that having a multiplicity of duplicative patient records is not the aim; rather, the goal is to create a shared patient record using approved data sources that all services can access. Long-term care facilities such as care and nursing homes are essentially the residents’ homes, meaning that to be able to provide a homely and comfortable setting, a balanced approach regarding data collection needs to be achieved. On the one hand, having electronic health records as in a hospital setting would open up opportunities for more decision support tools that could potentially reduce the workload of staff and help to improve care quality. On the other hand, to offer as comfortable a living environment as possible to residents, data collection should be limited to only those occasions when residents are ill or are at risk of an illness.

To strike a balance, minimum data sets could be the answer. A review of uptake of minimum data sets by Musa et al [47] evaluated different contexts, mechanisms, and outcomes to describe why minimum data sets were used in care homes, including system-level, care home–level, and individual-level barriers and facilitators. Some of the barriers mentioned include frequent staff turnover, training issues, and lack of computer skills. Facilitators include clinical staff presence, inclusive and understanding care home culture, and clarity of roles in data collection. These are very similar to barriers and facilitators for the implementation of clinical decision support tools that were identified in the studies in this review. The factors associated with successful system implementation in clinical practice were as follows: involving the administrator or head of nursing in the process; engaging the leadership in the project; presence of an internal champion; and participation of an interdisciplinary team, facilitators, and quality improvement team. In addition, it was deemed necessary to consider clinical workflow and training needs. It was also recommended to have a longer evaluation period to assess the effect of clinical decision support systems [3].

### Conclusions

Overall, the studies demonstrate that decision support tools can show improvements in delivery of care and in health outcomes, specifically in relation to medication management, falls prevention, management of dementia, pressure ulcer prevention and management, and nutritional assessment and management. However, there is variability in results, in part because of the diversity of types of decision support tools, users, and contexts as well as limited validation of the tools in use and in part because of the lack of clarity in defining the whole intervention. An important aspect that the studies seem to highlight is that decision-making to support care home residents is not just about providing technology within care homes; it also requires an effective multiagency approach with interaction with the wider multidisciplinary team outside the care home and supportive organization and culture to embed the use of the decision support tools.

## Appendix

Multimedia Appendix 1 Data extraction table.

## Abbreviations

DELIRIUMS: drugs, eyes and ears, low oxygen states, infection, retention of urine or stool, ictal, underhydration or undernutrition, metabolic, and subdural
MNA: Mini Nutritional Assessment
PGx: pharmacogenetic
PRISMA: Preferred Reporting Items for Systematic Reviews and Meta-Analyses
RAPS: Risk Assessment Pressure Sore
RQ: research question
SBAR: Situation, Background, Assessment, Recommendation
